# Bio-Availability, Anticancer Potential, and Chemical Data of Lycopene: An Overview and Technological Prospecting

**DOI:** 10.3390/antiox11020360

**Published:** 2022-02-11

**Authors:** Adriany das Graças Nascimento Amorim, Andreanne Gomes Vasconcelos, Jessica Souza, Ana Oliveira, Beatriz Gullón, José Roberto de Souza de Almeida Leite, Manuela Pintado

**Affiliations:** 1 Rede Nordeste de Biotecnologia, RENORBIO, Campus Ministro Petrônio Portela, Universidade Federal do Piauí, UFPI, Teresina 64049-550, PI, Brazil; 2 Núcleo de Pesquisa em Morfologia e Imunologia Aplicada, NuPMIA, Área de Morfologia, Faculdade de Medicina, Universidade de Brasília, UnB, Brasilia 70190-900, DF, Brazil; andreannegv@gmail.com (A.G.V.); jrsaleite@gmail.com (J.R.d.S.d.A.L.); 3 Centro Universitário do Distrito Federal, UDF, Brasília 70390-045, DF, Brazil; 4 People&Science, Brasília 70340-908, DF, Brazil; 5 Laboratório de Cultura de Célula do Delta, LCC Delta, Universidade Federal do Delta do Parnaíba, UFDPar, Parnaiba 64202-020, PI, Brazil; jessicamtsouza@gmail.com; 6 Laboratório Associado, Centro de Biotecnologia e Química Fina, CBQF-ESB, Universidade Católica Portuguesa, 4169-005 Porto, Portugal; asoliveira@ucp.pt (A.O.); mpintado@ucp.pt (M.P.); 7 Departamento de Ingeniería Química, Facultad de Ciencias, Campus Ourense, Universidad de Vigo, As Lagoas, 32004 Ourense, Spain; bgullon@uvigo.es

**Keywords:** anti-inflammatory, bio-availability, cancer, lycopene, nanostructure

## Abstract

The purpose of this review was to collect relevant chemical data about lycopene and its isomers, which can be extracted using different non-polar or polar aprotic solvents by SC-CO_2_ or biosynthesis as a friendly technique. Lycopene and other carotenoids can be identified and quantified by UV–Vis and HPLC using a C18 or C30 column, while their characterization is possible by UV–Vis, Fluorescence, FTIR, MS, NMR, and DSC assays. Among these techniques, the last four can compare lycopene isomers and identify *cis* or *all-trans*-lycopene. FTIR, MS, and NMR techniques are more suitable for the verification of the purity of lycopene extracts due to the signal complexity generated for each isomer, which enables identification by subtle differences. Additionally, some biological activities of lycopene isolated from red vegetables have already been confirmed, such as anti-inflammatory, antioxidant, and cytotoxic activity against cancer cells, probably by activating several pathways. The encapsulation of lycopene in nanoparticles demonstrated an improvement in oral delivery, and ex vivo assessments determined that these nanoparticles had better permeation and low cytotoxicity against human cells with enhanced permeation. These data suggest that lycopene has the potential to be applied in the food and pharmaceutical industries, as well as in cosmetic products.

## 1. Lycopene Bio-availability

**Figure 1:**
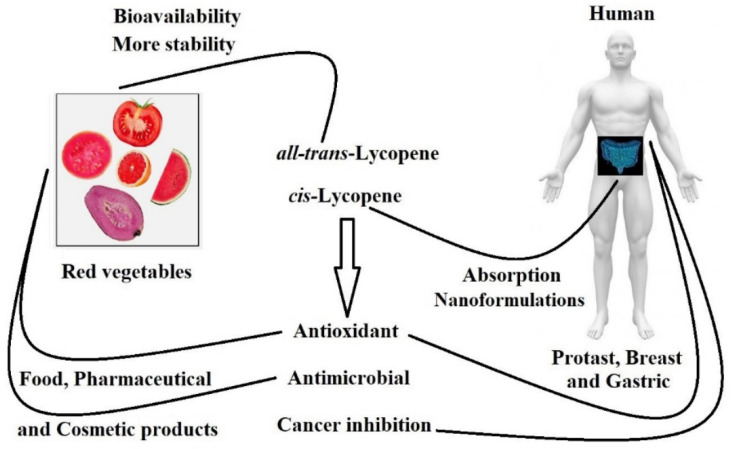
Representation of bio-availability and functions of lycopene in humans as well as products from foods, pharmaceutical and cosmetic products.

Lycopene is a carotenoid abundantly found in red vegetables (Figure 1). This natural pigment displays an important role in human biological systems due to its excellent antioxidant and health-supporting functions, which show a protective effect against cardiovascular diseases, hypertension, cancers, and diabetes [1,2].

Furthermore, studies have shown the potential anticancer activity of lycopene, suggesting that its consumption may prevent prostate, esophagus, stomach, colorectal, pancreas, breast, and cervix cancers. However, the literature has not determined the cause–effect relationship and how the consumption of lycopene-rich food decreases cancer risk [3,4,5,6].

Lycopene is an organic molecule whose molecular formula is C_40_H_56_ and molecular weight is 536.85 g·mol^−1^. It is insoluble in water but soluble in some organic solvents [7,8]. Its molecular structure contains 13 double bonds, 11 of which are conjugated and provide characteristics for lycopene’s antioxidant activity and strong red color [7,9].

These double bonds are affected by the action of oxidants, and can be damaged by light, acid, and heat, which destroy or rearrange the structure of lycopene to different spatial *cis* configurations from *all-trans*-isomers [10]. These effects may deteriorate and/or lead to the loss of lycopene bioactivity [5,10]. The latter seems to depend on several factors, including lycopene content, the complex composition of food, and particle size consumed in the digestive process [4].

**Figure 2:**
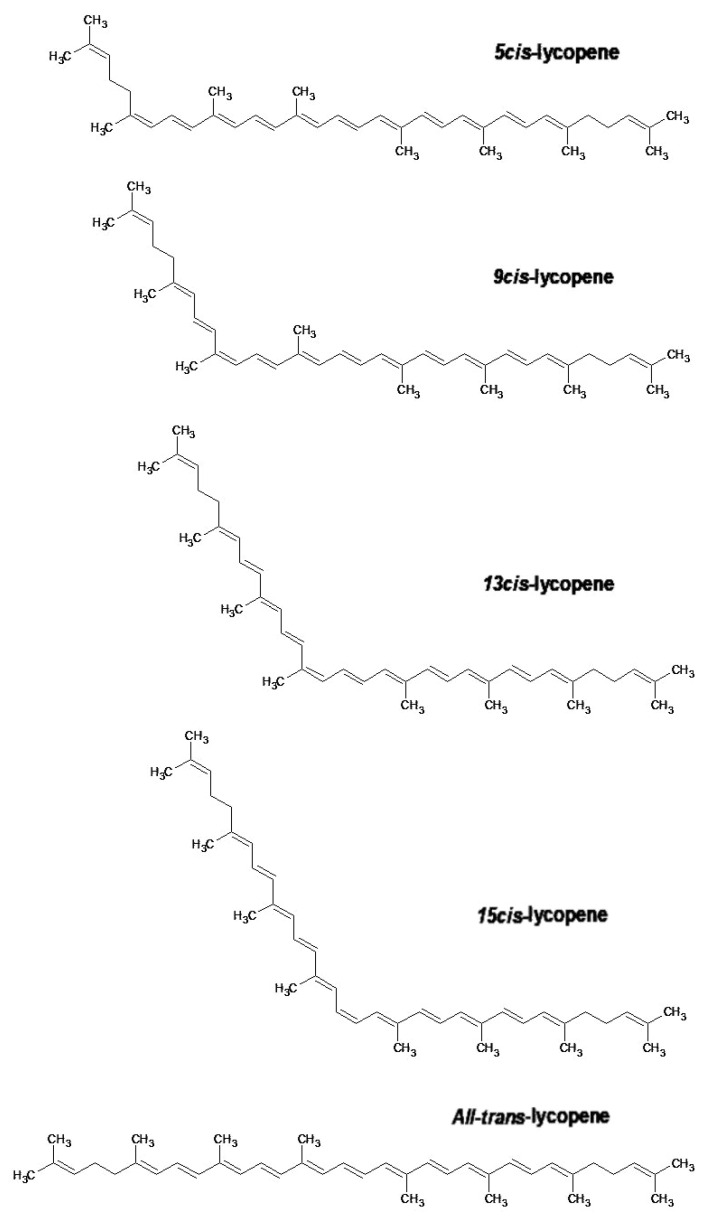
Representation of *cis* and *trans-*lycopene structure. Data deposited in computer by PubChem [14] and converted to 2D with ACD/Labs [15].

*All-trans*-lycopene (Figure 2) is interesting for industrial use in food and pharmaceuticals because of its high stability compared to other isomers of lycopene. *All-trans*-lycopene presents higher color intensity than *cis* isomers due to its low extinction coefficient. In nature, the *trans*-lycopene configuration has better stability compared with the *cis* isomer [3]. Nevertheless, the industry has also demonstrated interest in *cis-*lycopene structures (Figure 2) because they seem to have better bio-availability when compared to the *all-trans* isomer and can also prevent breast and prostate cancer [11,12,13].

The nutritional efficacy and industrial applicability of lycopene is limited due to its insolubility in water. On the other hand, nano-emulsified or micro-encapsulated lycopene, as well as lycopene nanoparticles, have demonstrated great in vitro bio-accessibility throughout the liberation of lycopene content from the nanostructure [3,11,16,17]. Therefore, micro- or nano-encapsulated lycopene may both overcome and avoid the problems related to lycopene structure and can present opportunities for the use of this potent antioxidant.

## 2. Production and Extraction Process

Several processes for the production and extraction of carotenoids such as lycopene have been proposed (Table 1). The most used method for extraction is solvent extraction and supercritical fluid extraction—SFE (supercritical CO_2_) [3,18,19]—and for production the most used is biosynthesis in a reactor or flask fermentation using biological strains, such as bacteria and yeasts [1,8,20,21]. Regardless of the method used, the lycopene extracts obtained have a red color, and their color intensity depends on lycopene concentration in the extraction media [22].

High temperatures (above 80 °C), light, oxygen, and exposure time may degrade lycopene, while the type of solvent can increase the isomerization from *all-trans*-lycopene to *cis*-lycopene. Acetone, for example, is one of the best solvents for extracting lycopene from fresh material once it provides better solubilization of the lipophilic intracellular content [26].

The use of electric processes to extract carotenoids from food has been successfully applied, with the advantage of promoting a selective extraction and improving carotenoid bio-availability [22]. Although the effects of electric processes on carotenoids are still unknown, applying low voltages could reduce the risk of damage to their structures [26]. These results have ushered in new studies about the factors that influence degradation, as well as “green” methods of lycopene recovery.

Supercritical CO_2_ is a technique that significantly impacts lycopene extraction, as it is considered an environmentally friendly method when compared with those that use solvents and lower temperatures [1,18,19,26]. The bacteria and yeasts introduced into bioreactors and the species *Escherichia coli, Blakeslea trispora,* and *Saccharomyces cerevisiae* stood out for lycopene production [8,20,21]. These microorganisms consume glucose, lactic acid, and fatty acids as nutrients at a temperature of around 30 °C, producing *β-*carotene and lycopene as final products [1,21].

Therefore, biosynthesis methods followed by ultrasound or enzymatic lysis to damage the cell membrane before supercritical fluid extraction could be an alternative to obtaining lycopene using a clean methodology.

It is noteworthy that the extraction methods applied in obtaining lycopene from red guava are patented methodologies and, for this reason, these methods are not mentioned in Table 1.

## 3. Chemical Characterization

### 3.1. UV–Vis

UV–Vis spectrophotometry has been employed as an efficient technique to characterize lycopene isomers (Table 2). In chloroform:ethanol (1:20), lycopene from red guava showed three absorbance peaks (λmax) at 501, 471, and 444 nm, consistent with those obtained for the lycopene standard from tomato (504 nm, 474 nm, and 447 nm) [13].

The characterization of lycopene extracts in different organic solvents, such as hexane:acetone, ethyl lactate, acetone, chloroform, and ethanol, demonstrated maximum absorption wavelengths at 470 nm, 478 nm, 473 nm, 473 nm, and 472 nm, respectively [3,11,16,26]. In other words, λmax of the lycopene spectrum depended on the solvent used for its solubilization [3,26,28].

### 3.2. Fluorescence

Fluorescence is another spectral technique successfully used to characterize loaded lycopene. Spectra have shown an excitation wavelength (λex) at 365 nm for lycopene-loaded *Chlorella pyrenoidosa* cells [10] and lycopene distribution in strains exhibiting at 488 nm in the absence and presence of fluorescence λex [21].

Lycopene analysis at pH 7.0 and pH 8.1 had a red-shift with λex from 334 nm to 338 nm and 335 nm to 338 nm, respectively. When associated with β-lactoglobulin at pH 7.0, lycopene presented λex of 295 nm [29]. Different fluorescence emissions were also observed for lycopene-loaded hydrolyzed collagen particles, which presented maximum fluorescence λex at 627 nm for red, maximum λex at 592 nm for orange, maximum λex at 522 nm for green, and maximum λex at 478 nm for light blue [30].

### 3.3. High-Performance Liquid Chromatography (HPLC)

Chromatograms obtained using C18 and C30 columns have shown different retention times (Rt), between 8 min to 20 min, for the *all-trans*-lycopene standard and its isomers [11,12,26,27,28]. The literature (Table 2) also reports different run times for these columns using a mobile phase in gradient or isocratic mode at a flow run of 1 mL·min^−1^ or 3 mL·min^−1^ [6,11,16,26].

The signs for undefined carotenoids reported for samples obtained from tomato byproducts and red guava were found at lower and higher Rt compared to the Rt reported for *all-trans*-lycopene [12,26]. Both red guava and tomato extracts demonstrate Rt for *5cis*-lycopene and *13cis*-lycopene close to the Rt of *all-trans*-lycopene [11,16,26,28]. However, the Rt of *15cis*-lycopene obtained from fine crystalline powder was higher than *β-*carotene [31].

Most studies analyzed in this review used the lycopene standard sold by Sigma–Aldrich, which contains ≥ 90% purity and consists of the sum of *trans* and *cis* isomers. This standard enables the detection of *15cis, 13cis, 9cis, 5cis* isomers, and *all-trans-*lycopene [6,16,19,23,28]. Thus, the use of solid–liquid extraction followed by purification methods and chromatography seems to be efficient to identify and quantify lycopene in extracts from red fruit [28,32,33].

### 3.4. Fourier Transform Infrared (FTIR)

The literature reports differences between *all-trans*-lycopene theoretical FTIR and its isomers. Signs at 2931 cm^−1^ and 1233 cm^−1^ are absent on the theoretical spectra of *5cis*-lycopene and *13cis*-lycopene, respectively. However, these signs were detected in the FTIR spectra of guava lycopene ethanolic extract, as well as in the theoretical FTIR *all-trans*-lycopene [13]. Additionally, lycopene spectra presented characteristic bands at 959 cm^−1^ or 960 cm^−1^, indicating a characteristic of out-of-plane C–H bending mode for a *trans*-conjugated alkene [2,28].

In general, the vibrational spectral pattern that can be attributed to both isomers of lycopene contain the highest wavenumbers at 2846, 2848, 2856, 2916, 2920, and 2927 cm^−1^ associated with C–H stretching and an absorption band of alkenyl C=C at 1633 cm^−1^. Bands from 1746–1229 cm^−1^ are C–C stretching, C=C, and C–H angular modes in the full molecule [9,13,28].

However, *cis*-lycopene isomers in extracts produced other vibrational modes between 1454 or 1446 cm^−1^ and 1366 or 1365 cm^−1^, indicating a reliable lycopene C=C stretching and an absorption band of alkenyl C–H (sp^2^ stretch) at 3043 cm^−1^ [9,13,28].

### 3.5. Mass Spectrometry

LC-MS/MS triple quad mass and mass spectrometry/ESI source in the positive mode can be used for the identification of the lycopene isomers in red extracts (Table 2). The results reported in the literature showed the presence of two molecular ions, [M]^+^ and [M+1]^+^, for lycopene [9,28,34].

After an experiment of collision-induced dissociation (CID), four main ions arose at *m*/*z* 467, 444, 375, and 269 for *all-trans*-lycopene [13,28]. Moreover, *5cis*-lycopene isomers presented one more fragment at m/z 309 generated by the loss of 227 Da from fragmentation patterns of ion m/z 536 [28]. By contrast, the loss of 15 Da (CH_3_) by CID of the same fragmentation pattern produced an ion of *m*/*z* 521, characteristic ions of *13cis*, and *15cis*-lycopene isomers [34].

In other words, mass spectrometry (MS/MS) can identify lycopene isomers because the collision-induced dissociation experiment is based on the fragmentation of mass-selected primary ions, generating different fragmentations between the molecules of lycopene isomers [28].

### 3.6. Nuclear Magnetic Resonance (NMR)

Methods such as NMR and FTIR can reveal the extent of impurities of lycopene crystals by analyzing their peaks with and without isomerization signs and comparing them to the peaks of a lycopene standard [13,28]. The structures of purified *cis*-lycopene could be assigned according to those of the *all-trans*-lycopene by ^1^H and ^13^C NMR spectroscopic analyses [35].

Both *5cis* and *15cis*-lycopene had nine and four different chemical shift values, respectively, from those presented for the *all-trans-*lycopene [28,31]. Moreover, Amorim et al. (2018) [28] showed that 45% of all carbons from the molecule of lycopene from red guava were compatible with the theoretical values of the ^13^C chemical shift of *5cis*-lycopene isomer.

Theoretical NMR was also used to confirm the type of lycopene obtained from purification methodology, aiming to know the type of lycopene isomer formed in the final process [28]. This process was possible because the lycopene crystals were produced by purification in HPLC and under all precautions taken for its storage, protecting it from air and light exposure [13,28].

### 3.7. X-ray Diffraction (XRD)

The crystallinity degree of lycopene samples has been defined by XRD patterns (Table 2). The diffractograms of lycopene samples obtained from red guava present intense and well-defined peaks at 2θ values of 12.38° and 12.42°, as well as broad peaks at 23.62° and around 22° and 25°. These values indicated that this type of lycopene had a degree of crystallinity greater than 70% [16,28].

The XRD patterns found for encapsulated lycopene also showed four sharp peaks but at 2θ values greater than 16°, which is characteristic of diffractions for a good crystal structure [10]. In the same way, the pattern observed for the *all-trans*-lycopene showed six diffraction peaks at similar 2θ values demonstrating that the powder of this carotenoid also has excellent crystallinity [6].

A nano-emulsified system involving lycopene purified from red guava demonstrated sharp peaks in the XRD patterns [10,16,28]. However, lycopene nano-emulsions have shown diffuse diffraction patterns, which are characteristic of their amorphous state [16]. Additionally, the diffraction peaks for lycopene crystals are not detected in encapsulated lycopene [6].

### 3.8. Differential Scanning Calorimetry (DSC)

Thermogravimetric curves of nano-encapsulated lycopene obtained by DSC can provide helpful information to elucidate the thermal stability of these nanostructures [10].

DSC analysis showed the successful encapsulation of lycopene in nano-emulsion droplets. The results indicated a sharp peak around 170 °C, which corresponded to the lycopene melting point, with good crystallinity degree. However, the nano-emulsion containing lycopene did not show the same melting point, showing an amorphous structure in the hydrophobic area of the nano-emulsion, where lycopene was located [2].

Nonetheless, a DSC curve for a lycopene nano-emulsion presented inflections at three points, while purified lycopene had two inflection points, indicating that the transition between polymorphic and crystal melting occurred [16]. DSC peaks at 398 °C and 430 °C also indicated the degradation of the nano-encapsulated or nano-emulsified lycopene after heating [10,16]. Accordingly, the nano-emulsion provided more thermal stability for lycopene and was more effective in preventing or minimizing the thermal-oxidative damage to lycopene [16].

Likewise, the DSC of cellulose acetate films containing lycopene showed an endothermic event, and this may occur close to a temperature that depends on the substitution degree of cellulose acetate [36].

## 4. Quantitative Analysis

### 4.1. Extraction

The combination of reverse osmosis with the diafiltration process generated a good yield of lycopene from tomato extract. On the other hand, when using a hexane:acetone:ethanol solution to produce a tomato extract, the initial content of lycopene tripled [37].

The initial content of lycopene from dried tomato peels found by Kehili et al. (2019) [24] was similar to the content of lycopene produced by reverse osmosis, which was extracted using oil at 2.5% (*w*/*v*), at high room temperature, and under magnetic stirring. Nevertheless, the quantification of an extract obtained by the Soxhlet method showed that the fraction from tomato seeds contained 37 times less lycopene than the fraction obtained from dried peels [24].

Moreover, pulsed electric field (PEF) treatment improved the extraction rate and the lycopene percentages in acetone and ethyl lactate. However, acetone extraction provided the highest yield for *all-trans-*lycopene [26].

Quantification methods showed that it might be possible to remove the lycopene content from fresh tomato byproducts without resorting to organic solvents [22], demonstrating a high yield of lycopene extracted by ultrasound-assisted processes and greater efficiency of lycopene encapsulation [3].

Definitively, thermal treatments have produced more lycopene than ultrasound extraction. Although both methods provide *cis* and *trans*-lycopene, *5cis*-lycopene is obtained in higher quantities when compared with *cis* isomers found in tomato peel [27].

### 4.2. Biosynthesis

Results of biosynthesis showed that engineered strains of *S. cerevisiae* (Table 1) produced good lycopene per dry weight (DW) by fermentation, with a relative increase reported by original high-yield *S. cerevisiae*. This method produced the highest yield reported to date in units of g·L^−1^ and mg·g^−1^ of DW [21].

Furthermore, mutant strains of *E. coli* produced more lycopene than the control starting the fermentation process with short-chain lipopolysaccharide. However, in some cases, the control supplied more lycopene than other strains of *E. coli*. The maximum yield for lycopene production was obtained by individual or combined strains of *E. coli*, indicating that the use of a smaller lipopolysaccharide might increase the lycopene biosynthesis because it increases membrane permeability [20].

Liu et al. (2020) [1] reported that biosynthesis systems containing *E. coli* strains improved the synthesis of lycopene from fatty acids in bioreactors fed with glucose and waste cooking oil under several hours of fermentation. This biomass contained lycopene corresponding to the highest amount reported to date for an engineered *E. coli* strain.

### 4.3. Supercritical Carbon Dioxide (SC-CO_2_)

Extraction methods by SC-CO_2_ have obtained *cis*-lycopene and, after centrifugation, could obtain a high *cis*-lycopene amount. On the other hand, solvent extraction could provide lower lycopene when using hexane for the process [19]. Thus, the SC-CO_2_ technique could increase the extraction efficacy to obtain lycopene simply and cleanly [18,19].

The highest yield obtained for tomato lycopene extraction was achieved by applying methodological conditions of sample preparations on peel/seeds ratio, using temperature and pressure, which reduced lycopene waste, making a correlation with the coefficient of mass transfer and an inverse connection with the solute partition coefficient [23].

### 4.4. UV–Vis and HPLC

Spectrophotometric methods have been used to determine the content of total carotenoids and lycopene previously dissolved in solvents such as hexane, acetone, ethanol, chloroform, or their mixtures [3,37]. The wavelengths used to measure lycopene content in samples were: 470 nm (hexane), 471 nm (chloroform:ethanol), 472 nm (ethanol), 473 nm or 474 nm (acetone), 478 nm (ethyl lactate), and 503 nm (n-hexane:acetone, hexane:acetone:ethanol). As we can see, the wavelengths for the detection of lycopene by spectrophotometry vary according to the solvent used [1,3,11,13,26,37,38].

Nano-emulsions containing lycopene were also quantified with the aid of calibration curves from UV–Vis but using wavelength detection at 473 nm [16]. Other lycopene nanoformulations verified the stability and quantified the lycopene at 457 nm [10]. The initial amount of lycopene micro-encapsulated with maltodextrin was quantified by the same technique at 503 nm [10,16,37]. We can say that the detection and quantification occur at a wavelength of maximum absorption within UV–Vis, both by the lycopene in the matrix as the organic solvent that solubilizes it.

Commercial *all-trans-*lycopene, when dissolved in organic solvents such as acetone or ethyl lactate, generated external calibration curves with concentrations ranging from 10 to 100 mg·L^−1^ and acceptable linearity (R^2^ ≈ 0.99) for both solvents. The lycopene content expressed in the extracts was twice as large in acetone than ethyl lactate for dry material [26]. In other words, HPLC analysis suggested that acetone was more effective in extracting *all-trans*-lycopene of wet peels from tomato than ethyl lactate.

The separation and/or quantification of lycopene and its isomers can be carried out by HPLC operating at high pressure, a binary gradient system, and a photodiode array detector [16,18,22,24,25]. It is important to know that lycopene isomers can be easily separated by chromatography using a reverse-phase column, which enables the quantification of each isomer, employing the *all-trans-*lycopene standard as a reference. The content of *cis*-lycopene isomers can be assessed by evaluating the relative peak area using the *all-trans*-lycopene as an external standard in HPLC calibration curves [6,28].

The lycopene content detected in tomato corresponded to the yielding calculated for the amount of dry mass as reported in the literature [18]. HPLC also showed the mean content of total carotenoids in mg of *β-*carotene. 100 g^−1^ of dry weight (DW), as well as the highest amounts of lycopene (also found in DW), represented the main carotenoids obtained in the by-product of the tomato production chain [25].

In addition, it is possible to identify lycopene *cis* isomers in samples, equating their Rt by HPLC coupled with a DAD detector and UV–Vis absorption spectra with lycopene standards [16,27,28]. UHPLC has also been reported as an adequate technique to identify and quantify *5cis*, *9cis*, 13*cis*, and *15cis* isomers [27]. Among these, *5cis* seems to be the most abundant lycopene *cis* isomer obtained from natural sources [27,28].

## 5. Antioxidant Activity

A few studies show the antioxidant activity of lycopene. The methods usually applied to determine the antioxidant potential of lycopene, such as ABTS, FRAP, DPPH, and ORAC have not been validated yet because they present difficulties in working with purified extracts [13,16,28].

The FRAP (ferric reducing antioxidant power) method has been used to evaluate the antioxidant potential of lycopene extracts from tomato peels in acetone and ethyl lactate. It was observed that PEF treatments significantly enhanced the antioxidant potential of *all-trans*-lycopene by 18.0% and 18.2%, respectively, when compared with untreated samples [26].

The antioxidant power of lycopene was also calculated by ABTS and ORAC assays. Data showed good linear regression coefficients (R^2^ > 0.9) for the analysis of lycopene extract and purified lycopene [13,16,28]. Moreover, ORAC results demonstrated that red guava had higher antioxidant levels than tomato, with particularly high antioxidant activity observed for the extracts instead of purified lycopene. These results may be associated with the mixture of carotenoids in the extract, enhancing its antioxidant capacity [28].

TEAC values obtained for the purpose of defining the antioxidant power of lycopene also demonstrated good correlations (R^2^ = 0.93) through simple linear regression analyses, which seems to suggest that lycopene may display good scavenging capacity towards ABTS^•+^ [39].

The antioxidant activity of a lycopene nano-emulsion was also assessed by analyzing its capacity to scavenge ABTS^•+^ compared with Trolox, with results of 127.66 ± 0.45 mM Trolox.g^−1^. The results were relatively lower when compared with purified lycopene and corroborated the results previously published for lycopene nano-emulsion [16].

Consequently, lycopene nano-emulsions with particle sizes between 100 and 200 nm subjected to DPPH and ABTS^•+^ assays demonstrated higher antioxidant activities than particles with smaller size, and this phenomenon may be due to the high dispersibility of these lycopene particles in the nano-emulsions, which makes them efficient against free radicals [40].

## 6. Antimicrobial Activity

Lycopene treatment caused stages of apoptosis in fungal cells, increased intracellular peroxyl radicals, intracellular mineral overload, and mitochondrial dysfunction (Table 3), resulting in cell death of *Candida albicans* strains [41].

Controlled release experiments using carotenoid-loaded poly DL-lactide-coglycolide nanoparticles showed that the slower carotenoid release rate inhibited *Listeria innocua* growth according to the concentrations tested, while the free carotenoid extract did not show the same behavior [42].

In this regard, lycopene inhibited and prevented the growth of relevant foodborne bacteria strains. A study showed that tomato peel oleoresin containing 2% of lycopene stopped the growth of both Gram-positive and Gram-negative bacteria and exerted antifungal effects by inducing apoptosis and cell dysfunction [43].

Additionally, lycopene extract showed antibacterial efficacy against *Escherichia. coli*, *Staphylococcus aureus,* and *L. innocua*, with MBC values of 20 mg·mL^−1^, suggesting that lycopene extract had potential applicability for the industry, with multifunctional characteristics in food, cosmetics, and pharmaceuticals [28].

Minimally processed apple containing lycopene in the form of microspheres showed an excellent microbial control quality, without contamination for *Salmonella* spp., *Listeria monocytogenes,* and generic *E. coli* for nine days. Moreover, inserting these microspheres at the highest concentration controlled the microbial growth of all these groups and increased the bioactive quality of the product (Table 3). By contrast, *Enterobacteria* loads did not change during food storage [27].

Lycopene extracts from tomatoes also exhibited activity against the growth of Gram-positive bacteria, which had a good correlation with the amount of isochlorogenic acid in tomatoes [25]. These results (Table 3) suggest that lycopene from guava and tomato correspond to bioactive molecules of natural sources with good microbial performance.

## 7. Anti-Inflammatory Assays

Guava lycopene extract presented good results against acute inflammation. The oral and intraperitoneal administration of this extract effectively subdued inflammation, reducing the impact of different phlogistic agents used to cause edema formation. Leukocyte migration inside paw muscle tissue, in the peritoneal cavity, and the MPO concentration were reduced, resulting in the protection of muscle tissue against oxidative stress effects and inhibiting gene expression involved with the down-regulation of inflammatory mediators [44].

## 8. Anticancer Pathways

The powerful antioxidant capacity of lycopene neutralizes reactive oxygen species and decreases oxidative stress. For this reason, lycopene consumption has been associated with a decreased rate of cancer development (Figure 1). Studies have demonstrated the potential of lycopene to inhibit several types of cancer, such as prostate, stomach, skin, and breast cancers [5,11,12,13,29].

Mechanisms involving the decrease in cancer risk by consuming lycopene-rich food are still unclear, even knowing that *all-trans* and *5-cis* isomers were already found in human tissues. However, the literature suggests that higher consumption of this type of food may reduce the risk not only for cancer but also for cardiovascular diseases, and boost the immune system [3,4,5,6].

Current evidence also suggests that the protection offered by lycopene against some types of cancer may be associated with its capacity to reduce cholesterol, inhibit oxidation processes, modulate inflammatory signs, enhance intercellular communication, stop tumorigenesis, and promote apoptosis, besides displaying antiangiogenic effects [45].

The investigation of the cytotoxic activity of lycopene against different types of cancer cell lines showed that concentrations from 50 to 100 µM of lycopene caused 42% and 43% of cell growth inhibition, respectively. This inhibition may happen via apoptosis induction and by impeding the expression of growth and survival genes [46].

Lycopene also reduced intracellular and mitochondrial free radical levels by decreasing the activation signal-regulated kinase pathways, attenuating the DNA-binding activity and the expression level of the COX-2 gene AGS cells [47].

Moreover, the investigation of the anticancer activity of lycopene against human breast cancer cells showed that the lycopene extract notably affected the viability of these cells. This study demonstrated that lycopene reduced cell proliferation, generated cell cycle arrest, and promoted modifications in the mitochondrial membrane and DNA fragmentation, as well as exhibiting no hemolytic activity and low cytotoxicity against peritoneal macrophages [13].

Lycopene nanoparticles also notably decreased the growth of MCF-7 cells, causing more than 50% growth inhibition after one and three days of treatment, even at lower concentrations. It was possible to see that the nano-encapsulation enhanced its toxicity against these cells when compared with the free lycopene. Moreover, these lycopene nanoparticles inhibited the activation and production of free radicals in microglial cells, without affecting the membrane of erythrocytes. Thus, these nanoparticles may have the physicochemical and biological potential for application as health-promoting nutraceuticals and in cancer treatment [12].

In addition, lycopene nanoparticles synthesized with modified cashew gum optimized the cytotoxic effect of the free lycopene extract against breast cancer cells, presenting biocompatibility with keratinocytes and erythrocytes. The nanoparticles were non-toxic to *Galleria mellonella* larvae, which demonstrated their low toxicity despite the anticancer potential [11].

These results mean that lycopene is a safe product, with anticancer potential against the human gastric, breast, and prostate cancer cells, via different pathways with no harm against normal human cells. However, the pathways involved with its mechanism of action still need to be clarified.

## 9. Lycopene Bio-accessibility and Bio-availability—Novel Technologies

Carotenoids can offer numerous health benefits when consumed consistently (Table 4). Nevertheless, for this purpose, there must be a first release from the food matrix followed by carotenoid diffusion into oil droplets. The bile salts help to create micelles to assist the digestion of lipid forms, converting them in free fatty acids, mono- and diacylglycerides, lysophospholipids, and free cholesterol [48]. The harsh conditions throughout the absorption and assimilation process might conduct lycopene degradation due to exposure to pH changes, increased temperature, and oxidation [49]. Consequently, bio-availability and absorption are much lower than water-soluble molecules [50].

Strategies have been created to control problems related to the practical application of lycopene, which is strongly restricted due to its high sensitivity when exposed to light, oxygen, and heat, as well as contact with metal ions, besides processing conditions and low water solubility [11,51].

Encapsulation is one of the techniques that frequently uses oligosaccharides, such as cyclodextrins, to associate compounds in a hydrophobic core, while on the outside it forms a hydrophilic shell. The encapsulation protects lycopene from degradation and isomerization besides increasing its solubility in aqueous environments [51]. A report on the lycopene/*α-* and *β-*CD complexes pointed that both could provide stable associations in water with profound differences in structure [66]. Maltodextrins were used during tomato processing to powders aiming to increase lycopene stability [67].

Respective delivery systems have been created to enhance lycopene bio-availability and absorption rates (Figure 1) in the gastrointestinal environment [57,68]. During digestion, carotenoids are assimilated with other lipids into mixed micelles containing bile salts and phospholipids, which perform as carriers to solubilize the carotenoids and transport them to the zone of maximum absorption in the intestine. Incorporating lycopene into the oil phase of emulsions is an alternative to protect it from oxidation and chemical degradation, providing better bio-availability and prolonging shelf-life [48]. Nano-emulsions have been reported to be suitable delivery systems with favorable results for the encapsulation of low-solubility compounds, such as lycopene [51]. Recent works have shown efficient delivery systems for lycopene: its incorporation in oil-in-water emulsions for orange beverages [69], lycopene encapsulated in isolate-Xylo-oligosaccharide protein conjugates made by Maillard reaction [68], and oil-in-water emulsions with long- to short-chain triglycerides [57]. The literature also reports the formation of liposomes and nanoliposomes, which are spherical vesicles created with a concentric phospholipid bilayer of hydrophilic center. It was reported that lycopene tends to be entrapped in the hydrophobic bilayer, enhancing bio-accessibility when exposed to the gastrointestinal tract and with increased antioxidant capacity compared with the free form [70,71].

Oil–water nano-emulsions are nanoparticles dispersed in heterogeneous systems with an inner lipidic and an external aqueous phase stabilized by one or two surfactants. Unlike the nano-emulsions, lipid nanoparticles have an internal solid lipid phase since these nanoparticles are totally or mainly composed of solid lipids at room temperature. Such a solid matrix allows the controlled release of the encapsulated molecules and protects them from degradation while increasing the long-term stability of the system [72]. Lycopene-loaded SLNs demonstrated stability in an aqueous medium for two months, producing an applicable system for future in vivo trials in nutraceutical industries [59]. The encapsulation in SLN showed an improvement in lycopene oral delivery, and an ex vivo assessment determined that this carotenoid had better permeation besides causing more cytotoxicity against breast cancer cells [62]. Lycopene loaded into nanostructured lipid carriers (NLC) composed of Eumulgin SG, orange wax, and rice bran oil, employing high pressure in homogenization process, showed chemical stability and delayed degradation when put into cold storage [73].

Moreover, lycopene nano-emulsions have provided more thermal stability for lycopene and significantly inhibited edema formation. For this reason, these nanoparticles may be considered to be a potential candidate for anti-inflammatory therapy [16]. Lipid-core nanocapsules of lycopene, in turn, optimized stability for 7 months at 5 °C storage, and improved its toxicity against breast cancer cells. The nanocapsules also inhibited the production of intracellular peroxyl radicals in human microglial cells and maintained the membrane integrity of erythrocytes, highlighting its potential to be employed in cancer treatment [12].

Lycopene encapsulated in polymeric nanoparticles showed high anti-tumor potential, with cytotoxicity against cancer cells at low concentrations and no toxicity against *Galleria mellonella*. Additionally, nanoparticles with sizes of 162.10 ± 3.21 nm were efficient with a passive mechanism of permeability for targeting tumor tissues [11].

On the other hand, lycopene powder produced by complex coacervation and freeze-drying after microencapsulation had promising results as a biopolymeric composite with inhibitory effect potential on α-amylase associated with metabolic syndrome, and demonstrated high antioxidant activity in formulations [3].

## 10. Patent Databases

As shown in Table 5, a total of 180,796 patents were deposited in the Portuguese (INPI) [74,75], American (USPTO) [76], and European (EPO) [77] databases, as well as WIPO [78] (worldwide). The results showed that 0.009% and 0.058% of patents are registered in Portugal and Brazil (INPI database), 10.02% in USPTO, 41.24% in EPO, and 48.68% in WIPO. Currently, the terms “lycopene and guava”, as well as “lycopene and tomato” are more frequently used in the titles or abstracts of patents.

WIPO was the database where the highest percentage of patents relating to lycopene was registered, probably because it has become an international consortium where the global research community share their intellectual property and expertise, including private and public organizations.

The EPO patent filing database, in turn, is more complete and presents a source of publicly available procedural information on European patent applications. It was the second in the number of lycopene patents. By contrast, the lowest number of patents was found in INPI. The topic discussed suggests that there is little incentive to create intellectual property through lycopene and guava terms.

The data presented in this prospection table showed that the application of lycopene from red guava and its nanoformulations are still innovative, since there are few patents found when the term “lycopene and nano-emulsion and guava” was used.

## 11. Conclusions

Several types of *cis*-lycopene can be separated, identified, and quantified by HPLC. Analytical techniques such as UV–Vis, Fluorescence, FTIR, MS, NMR, and DSC can also be used for lycopene characterization by comparing it with standards previously established.

Extracted, purified, and nanostructured lycopene had good antioxidant activity confirmed by ABTS, DPPH, and ORAC assays. These lycopene products were efficient as anti-inflammatory agents and demonstrated cytotoxic effects against different cancer cell lines, probably by an apoptotic pathway without affecting normal cells.

The good thermal stability of nanostructured lycopene enables its use as an antioxidant agent. This characteristic adds value to the application of lycopene in different industry sectors because temperature determines the thermodynamic behavior of molecules.

Moreover, lycopene loaded in nanostructures had better permeation than the extract and purified lycopene, causing low cytotoxicity against human cells. These data suggest a promising potential application of this carotenoid in the development of foods, cosmetics, and pharmaceuticals products.

## Figures and Tables

**Table 1:** Type of lycopene obtained from different extraction methods and biosynthesis from 2017 to 2021.

Technique	Solvent/Mobile Phase/Flow Rate	Device	Temperature/ Pressure/Time/ V/Hz/rpm	Lycopene Source/ Carotenoid Extracted	References
Extraction	Hexane	Soxhlet extractor	37 °C/6 h	Tomato peel and seed/ *cis* and *trans*-lycopene	[19]
	* SC-CO_2_/1 mL·min^−1^	SFT 110 extractor	40 and 80 °C/30 and 50 MPa/30, 45, 60, 90, 120, 180, 240 min		
	Hexane/Acetone/Ethanol (2:1:1)	Vortex	2 h		
	Washed in 0.1 M NaCl			Tomato peel and seed/ Lycopene and *β*-carotene	[22]
	OH pre-treatment		55 °C/1 min		
	Water/Ethanol (70%) (1:6 *w*/*v*) ^&^	Thermal extraction	55 °C/15 min		
	Water/Ethanol (1:6 *w*/*v*) ^&^ + OH solution				
	OH application	Ohmic heating (OH) technology (6–11 V·cm^−1^) pre-treatment	0–100 °C/30 min/ 60–280 V/ 25 kHz		
	* SC-CO_2_/1 L·min^−1^	SFT 110 extractor	60 °C and 40 MPa/ 30, 45, 60, 90, 120, 180, 240 min	Tomato peel and seed/ Highest *cis*-lycopene content	[23]
	Hexane	Soxhlet extractor/0.22 µm hydrophobic PTFE	12 h	Tomato peel and seed/ Lycopene	[24]
	Olive oil	Maceration (15 to 150 min)/Magnetic stirrer/Box–Behnken	40–80 °C/ 200–400 rpm		
	Methanol/Ethyl acetate/ Petroleum ether (1:1:1, *v*/*v*/*v*)			Tomato peel and seed from 10 varieties/ Lycopene, *β*-carotene, and lutein	[25]
	30% Methanolic potassium hydroxide		Room temperature /6 h		
	Saturated saline solution/ Diethyl ether/ Distilled water	Washed			
	Dry over anhydrous sodium sulfate	Rotary evaporator R-124	35 °C		
		PEF (pre-treatment)1,3; 5 kV·cm^−1^/0.012 kJ·kg^−1^, 0.160 kJ·kg^−1^, 0.475 kJ·kg^−1^/10 Hz/20 μs	20 ± 2 °C	Tomato peels/ *cis* and *all trans*-lycopene	[26]
	Acetone (1:40 *w*/*v*) ^&^	Extraction flask	25 °C/0–24 h /160 rpm		
	Ethyl lactate (1:40 *w*/*v*) ^&^				
	Ethyl acetate	Thermal extraction	75 °C/1 or 2 h	Plum tomato peels/ *cis* and *all trans*-lycopene	[27]
		Ultrasounds	Approximately 0 °C/30 min		
	Magnesium carbonate (20%) (sample/solution 1:1)	Orbital shaker	25 °C/2 h	Tomato peels/ Lycopene	[3]
	*n*-hexane/Acetone (3:1)	Ultrasonic	50 °C/30 min (10 times)		
		Centrifuge	10 °C/10 min		
		Reduced volume	40 °C/Low pressure		
**Technique**	**Substrate**	**Device**	**Biological Strain**	**Carotenoid Produced**	**References**
Biosynthesis	Isopropyl-*β*-d-thiogalactoside (IPTG) as inducer	Shaking flask (37 °C and 200 rpm)	*E. coli*	Lycopene	[20]
	Glucose	Shake flask (30 °C, 300–600 rpm)	*S. cerevisiae*	Lycopene	[21]
	Glucose + Glycerol	Shake flask (37 °C, pH = 7.2, 48 h)	*Escherichia coli* R122	Lycopene	[1]
	Glucose				
	Oleic acid	Bioreactor (37 °C, pH = 7.2, 48 h)	*Escherichia coli* FA03-PM		
	Glucose				
	Glucose + oleic acid + yeast extract				
	Glucose + waste cooking oil + yeast extract				
	Lactic acid	Flask fermentation (120 h)	*B. trispora* NRRL 2895 (+) and N6 (−)	Lycopene and *β*-carotene	[8]

* SC-CO_2_ = Supercritical Carbon Dioxide; (*w*/*v*) ^&^ = sample weight/solvent volume.

**Table 2:** Characterization and quantification methods for *cis* and *trans*-lycopene from 2017 to 2021.

**Technique**	**Mobile Phase**	**Device**	**Carotenoid Results**	**References**
High-performance liquid chromatography (HPLC)	Acetonitrile/Methanol/ Hexane/Dichloromethane /Ammonium acetate (55:22:11.5:11.5:0.02) (*v*/*v*/*v*/*v*/*w*) Isocratic performance	C30 RPAQUEOUS (5 μm; 4.6 mm × 250 mm) DAD detector	*all-trans-*lycopene, *5cis*, *13cis-*lycopene, and *β*-carotene	[28]
			*all-trans-*lycopene, *5cis*, and *13cis-*lycopene	[16]
			*all-trans-*lycopene, and other carotenoids	[12]
	Methanol/ Methyl butyl ether/ Ethyl acetate (5:4:1)	C30 YMC (5 μm; 4.6 mm × 250 mm)	*15cis, 13cis, 9cis, 5cis,* other *cis* isomers, and *all-trans-*lycopene	[19]
	A: Ethyl acetate B: Acetonitrile/water (90:10) Gradient performance	C18 Vydac 201TP54 C (250 × 4.6 mmm) + C18 pre-column DAD detector	Total carotenoids in *β*-carotene and lycopene	[22]
	Methanol/Methyl butyl ether/Ethyl acetate (50:40:10)	C30 YMC (5 μm; 4.6 mm × 250 mm)	*cis* and *trans*-lycopene	[23]
	Acetonitrile/ Dichloromethane (75:25; *v*/*v*)	C18 Eclipse XDB (3.5 μm; 4.6 mm × 250 mm) DAD detector	Lycopene	[24]
	A: Methanol/Water (98:2) B: Methanol/Water (95:5) C: MTBE Gradient performance	C30 YMC (3 μm, 250 × 4.6 mm) + C30 guard column (20 × 4.6 mm) Column heater at 20 °C DAD detector	*15cis, 13cis, 9cis, 5cis,* di *cis isomers,* and *all-trans-*lycopene	[27]
	A: Acetonitrile/water (9:1, *v*/*v*) + 0.25% Triethylamine B: Ethyl acetate + 0.25% Triethylamine Gradient performance	C18 Nucleodur 300-5 (5 μm; 4.6 × 250 mm) DAD detector	Lycopene, *β*-carotene, and lutein	[25]
	Methanol (27%)/ Acetonitrile (23%)/ MTBE (50%)	C30 YMC	*all-trans*-lycopene,*13cis*, *9cis*, and total *cis*-lycopene isomers	[6]
	Acetonitrile/Methanol (10:90, *v*/*v*)/9 mM TEA (Triethylamine)	C18 reverse-phase ODS2 (5 μm; 4.6 mm × 150 mm) DAD detector	*all*-*trans*-lycopene Undefined carotenoid	[26]
	A: Acetone/water (75:25, *v*/*v*) B: Acetone/Methanol (75:25, *v*/*v*) Gradient performance	C18 Zorbax (3 μm; 3 mm × 250 mm) DAD detector	Lycopene	[18]
	Acetonitrile/Methanol/ Hexane/Dichloromethane /Ammonium acetate (55:22:11.5:11.5:0.02, *v*:*v*:*v*:*v*:*w*)	RPAQUEOUS Develosil-C30 (5 µm, 4.6 × 150 mm)	Lycopene isomer Lycopene Other carotenoids	[11]
**Technique**	**Solvent**	**Device**	**Carotenoid Results**	**References**
UV–Vis	Chloroform:Ethanol (1:20)	UV–Vis spectrophotometer	*all-trans*-lycopene	[13]
	Chloroform:Ethanol	UV-1800 spectrophotometer	Lycopene	[16]
	Water		Lycopene	
	*n*-hexane:acetone (3:1)	Spectrophotometer	Lycopene	[3]
	Acetone	Spectrophotometer	Lycopene	[1]
	Acetone	V-650 UV–Vis spectrophotometer	Lycopene	[26]
	Ethyl acetate		Lycopene	
	Ethanol	UV–Vis spectrophotometer	Lycopene	[11]
**Technique**	**Pellet/ATR Accessory**	**Device**	**Carotenoid Results**	**References**
Fourier Transform Infrared (FTIR)	KBr	FTIR	Lycopene	[9]
	KBr	IRAffinity-1 spectrometer	*5cis*-lycopene	[28]
	KBr	FTIR-ATR	Lycopene	[2]
	Diamond crystal plate	FTIR-ATR	*all-trans*-lycopene	[13]
	KBr	FTIR spectrometer	*all-trans*-lycopene	[6]
**Technique**	**Collision Gas (Energy)/** **Gas Flow/Temperature /Flow Rate/Capillary Potential**	**Device/*m*/*z***	**Carotenoid Results**	**References**
Mass spectrometry (MS)	5–18 eV/180 °C/ 4 L·min^−1^/4 kV	MS/MS Mass spectrometer Electrospray source [M]^+^ *m*/*z* 50–3000	*5cis*-lycopene	[28]
			*all-trans*-lycopene	
	15 eV/180 µL·h^−1^ Nitrogen gas/200 °C/ 4 L·min^−1^/4.5 kV	Mass spectrometer ESI source [M]^+^ *m*/*z* 50–1500	*all-trans*-lycopene	[13]
		HRMS ESI [M]^+^ *m*/*z* 100–1000	Lycopene	[12]
**Technique**	**Solvent/Temperature**	**Device/ppm**	**Carotenoid Results**	**References**
	CDCl_3_	Discovery Studio 3.5 + B3LYP + 6-311G (d, p) Theoretical NMR + (GIAO)^z^ +TMS^a^	*5cis*-lycopene	[28]
		400 MHz ^1^H NMR/0–10 ppm		
		400 MHz ^13^C NMR/0–150 ppm		
	D_2_O/25 ± 0.5 °C	Self-diffusion ^1^H NMR Avance III 600 MHz	Lycopene	[2]
	CDCl_3_	^1^H NMR	*all-trans*-lycopene	[4]
**Technique**	**Å/kV/A**	**Device/2θ/Steps**	**Carotenoid Results**	**Reference**
X-ray diffraction (XRD)	0.154 nm/40 kV/40 mA	D_8_ XRD diffractometer/ 4°–45°/0.1° (4°/min)	Lycopene	[10]
	50 kV/100 mA	X-ray diffractometer/ 5°–50°/5 s/step	*cis*-lycopene isomers	[28]
	1.54 Å/45 kV/40 mA	D_8_ XRD diffractometer/ 5°–45°/2°/min	*all-trans*-lycopene	[6]
			*cis*-lycopene	
	50 kV/100 mA	X-ray diffractometer/ 3°–50°/5 s/step	*cis*-lycopene	[16]

(GIAO)^z^ = Gauge Invariance Atomic Orbital; TMS^a^ = Tetramethylsilane.

**Table 3:** Antimicrobial activity of lycopene or lycopene extracts from 2015 to 2021.

Extract/Structure	Microorganisms	Results	References
Lycopene (5 µg/mL) from tomato	*C. albicans*	Antifungal effects against *C. albicans* by inducing apoptosis via ROS production and mitochondria dysfunction.	[41]
Carotenoids within PLGA nanoparticles	*L. innocua* (NRRL B-33076)	The nanoparticles were effective in preventing *L. innocua* growth.	[42]
Lycopene oleoresin	*E. coli, S. aureus, Salmonella typhi, L. monocytogenes, Bacillus cereus*, and *B. licheniformis.*	Oleoresin can inhibit and prevent the growth of relevant foodborne bacteria.	[43]
Lycopene extracts from guava and tomato	*E. coli*, *S. aureus*, and *L. innocua*	Extract of lycopene presents MBC values of 20 mg·mL^−1^.	[28]
*cis*/*trans-*lycopene microsphere from tomato	*Salmonella spp.*, *L. monocytogenes*, and generic *E. coli*	The microbial quality of the food samples was not highly affected (< 0.8 log units) during the storage period after the incorporation of lycopene microspheres.	[27]
Lycopene extract from tomato	Gram (+): *S. aureus, B. subtilis*, and *L. monocytogenes*. Gram (-): *Pseudomonas aeruginosa, S. typhimurium, E. coli,* and *Klebsiella pneumonia*	Extracts of all cultivars were more effective against *S. aureus*, moderate antimicrobial activity against *K. pneumoniae, P. aeruginosa, E. coli,* and *S. typhimurium*, but Tiny Tim cultivar was the most effective against *S. aureus, B. subtilis, L. monocytogenes,* and *K. pneumonia*	[25]

**Table 4:** Recent examples of methods used to encapsulate lycopene from 2017 to 2021.

Delivery System	Encapsulation Method	Results	References
*β-*cyclodextrins	A mixture of methylene chloride solution of lycopene with ethanol at 37 °C.	Higher stability against oxidizing agents (AAPH and H_2_O_2_).	[52]
*β-*cyclodextrins	Lycopene inclusion complexes with *β-*cyclodextrin were prepared by the precipitation method.	Increased thermal stability, photostability, and antioxidant activity.	[38]
Nanoliposomes	Sonication of lycopene, soybean phosphatidylcholine, cholesterol, and aqueous solution.	Neuronal protection against cerebral ischemia/reperfusion. Improved therapeutic efficacy and attenuated the cardiotoxicity of the chemotherapy drug doxorubicin.	[53]
Phospholipid nanoliposomes	Nanospheres of phospholipids with lycopene produced by evaporation and nanoliposomes produced by sonication with the presence of buffer and recovered by centrifugation.	Enhanced antioxidant activity. Prevented reactive oxygen species-induced kidney tissue damage.	[54]
Double-loaded liposomes	Lycopene, *β-*cyclodextrins encapsulated with soy lecithin and cholesterol.	Prolonged-release. Improvement of lycopene solubility. Cardioprotective activity tested *in vivo.*	[55]
Oil-in-water nano-emulsions	Octenyl succinate anhydride-modified starch mixed with lycopene using high-pressure homogenization and medium-chain triglycerides as carrier oils.	Stable nano-emulsions system with potential application for functional foods.	[2]
Oil-in-water emulsions	Emulsion of water, pure whey isolate, citric acid, triglycerides, and lycopene created with pressure homogenizer.	Increased lycopene bio-accessibility. System critical for the delivery of lipophilic bioactive compounds in functional drinks.	[56]
Nanodispersions	Homogenization of lycopene dissolved in dichloromethane, aqueous phase, and Tween 20.	Small-size lycopene nanodispersions. Good stability for application in beverage products.	[57]
Feed emulsions	Homogenization of tomato powders, maltodextrin, and gum Arabic in aqueous solution and encapsulation made by spray-drying.	Increased lycopene stability.	[58]
Solid lipid nanoparticles (SLN)	Lycopene-loaded solid lipid nanoparticles using Precirol^®^ ATO 5, Compritol^®^ 888 ATO, and myristic acid by hot homogenization.	Stable after 2 months in an aqueous medium (4 °C).	[59]
Solid lipid nanoparticles (SLN)	Cold homogenization technique with glyceryl monostearate and lycopene.	Gel with a promising antioxidant therapy in periodontal defects.	[60]
Solid lipid nanoparticles (SLN)	Homogenization-evaporation technique of lycopene-loaded SLN with different ratios of biocompatible Compritol^®^ 888 ATO and gelucire.	Particles showed in vitro anticancer activity.	[61]
Nanostructure lipid carriers (NLCs)	Ultrasonication of lycopene with Tween 80 and Poloxamer 188.	Enhanced oral bio-availability. Increased cytotoxicity against human breast tumor cells.	[62]
Nanostructure lipid carriers (NLCs)	Homogenization and ultrasonication method (aqueous phase with Tween 80, lecithin, and lycopene).	Increased lycopene aqueous solubility. Improved solubility masking tomato aftertaste. Increased homogeneity of fortified orange drink.	[63]
Nanostructure lipid carriers (NLCs)	Emulsion created with lycopene, a lipid mixture, Tween 80 followed by pressure homogenization.	Biphasic release pattern with fast release initially and a slower afterward.	[6]
Whey protein isolate nanoparticles	Lycopene loaded whey protein isolate nanoparticles.	Enhance the oral bio-availability of lycopene. Controlled release. Facilitated absorption through the lymphatic pathway.	[17]
Gelatin nanofibers	A mixture of gelatin from bovine skin and tomato extract is used in electrospinning.	Better retention of lycopene. Better antioxidant activity during 14-days storage.	[64]
Ionic gelation	Lycopene watermelon concentrate mixed with sodium alginate or pectin. Encapsulation by dipping in CaCl_2_ and drying under vacuum.	More stable lycopene-rich beads. Good application as natural colorants/antioxidants in different types of food products.	[65]
Nano-encapsulation	CPCs (Chlorella pyrenoidosa cells) loaded with lycopene into a complex nutraceutical and exogenous.	Feasibility of lycopene encapsulation in the CPCs. Combined the activities of both materials. Novel nutraceuticals to reduce cellular oxidative stress.	[10]
Nano-emulsion	Lycopene from guava on nanoemulsifying system of natural oils.	Lycopene nano-emulsion with high stability. Significant inhibition of edema formation, suggesting a potential candidate for anti-inflammatory therapy.	[16]
Lipid-core Nanocapsules	Nano-encapsulation process mixed lycopene extract from guava with polycaprolactone polymer in acetone sorbitan monostearate.	The nanostructure was cytotoxic against cancer cells (human breast adenocarcinoma line MCF-7).	[12]
Nanoparticle	Polymer nanoparticle fucan-coated based on acetylated cashew gum and lycopene extract from guava.	Promising results for applicability in hydrophobic compounds carrying systems as lycopene with cytotoxic effect on the breast cancer cell.	[11]
Microencapsulation	Microencapsulation of lycopene from tomato peels by complex coacervation and freeze-drying.	The fine orange-yellow powder could be micro-encapsulated as stable lycopene applied to the food industry with properties against metabolic syndrome.	[3]

**Table 5:** Number of patents after search on INPI, EPO, USPTO, and WIPO databases (in columns) using the keywords inserted in Table 5 lines.

Keywords	INPI (Portugal)	INPI (Brazil)	USPTO (USA)	EPO (European)	WIPO (International)
Lycopene	11	58	5532	29,979	30,220
Guava	1	45	3315	14,051	18,396
Tomato	34	187	38,236	150,308	171,583
*Psidium guajava*	0	14	978	2007	4568
Lycopene and tomato	5	7	1290	8113	8390
Lycopene and tomato and extract	0	1	881	5265	7313
Lycopene and guava	0	2	235	1496	1467
Lycopene and guava and extract	0	0	207	1317	1355
Lycopene and nano	0	0	543	2638	2689
Lycopene and nano-emulsion	0	0	111	482	930
Lycopene and nano-emulsion and tomato	0	0	23	105	173
Lycopene and nano-emulsion and guava	0	0	21	31	81
Antimicrobial and Lycopene	0	0	1440	4018	7289
Antimicrobial and Lycopene and extract	0	0	1069	3054	6785
Antimicrobial and Lycopene and rich and extract	0	0	491	1192	2615
Antimicrobial and Lycopene and rich and extract and tomato	0	0	126	402	1026
Antimicrobial and Lycopene and rich and extract and guava	0	0	51	105	181
Total	51	314	54,549	224,563	265,061

Source: Patents databases INPI, EPO, USPTO, and WIPO on 7 January 2022. For INPI database searching, the following terms in Portuguese were also used: Licopeno; Goiaba; Tomate; *Psidium guajava*; Licopeno e tomate; Licopeno e tomate e extrato; Licopeno e goiaba; Licopeno e tomate e extrato; Licopeno e goiaba e extrato; Licopeno e nano; Licopeno e nanoemulsão; Licopeno e nanoemulsão e tomate; Licopeno e nanoemulsão e goiaba; Antimicrobiano e Licopeno; Antimicrobiano e Licopeno e extrato; Antimicrobiano e Licopeno e rico e extrato; Antimicrobiano e Licopeno e rico e extrato e tomate; Antimicrobiano e Licopeno e rico e extrato e goiaba.
